# Multi-level modelling of longitudinal child growth data from the Birth-to-Twenty Cohort: a comparison of growth models

**DOI:** 10.3109/03014460.2013.839742

**Published:** 2013-10-11

**Authors:** Esnat D. Chirwa, Paula L. Griffiths, Ken Maleta, Shane A. Norris, Noel Cameron

**Affiliations:** ^a^ Wits/MRC Developmental Pathways for Health Research Unit, Faculty of Health Sciences, University of the Witwatersrand JohannesburgSouth Africa; ^b^ Centre for Global Health and Human Development, Loughborough University, Loughborough Leicestershire LE11 3TUUK; ^c^ Department of Community Health, College of Medicine, University of MalawiBlantyre, Malawi

**Keywords:** Child growth, growth models, mixed effects modelling, structural and non-structural growth models

## Abstract

*Background*: Different structural and non-structural models have been used to describe human growth patterns. However, few studies have compared the fitness of these models in an African transitioning population.

*Aim*: To find model(s) that best describe the growth pattern from birth to early childhood using mixed effect modelling.

*Subjects and methods*: The study compared the fitness of four structural (Berkey-Reed, Count, Jenss-Bayley and the adapted Jenss-Bayley) and two non-structural (2nd and 3rd order Polynomial) models. The models were fitted to physical growth data from an urban African setting from birth to 10 years using a multi-level modelling technique. The goodness-of-fit of the models was examined using median and maximum absolute residuals, Akaike Information Criterion (AIC) and Bayesian Information Criterion (BIC).

*Results*: There were variations in how the different models fitted to the data at different measurement occasions. The Jenss-Bayley and the polynomial models did not fit well to growth measurements in the early years, with very high or very low percentage of positive residuals. The Berkey-Reed model fitted consistently well over the study period.

*Conclusion*: The Berkey-Reed model, previously used and fitted well to infancy growth data, has been shown to also fit well beyond infancy into childhood.

## Introduction

Human growth, like most developmental processes, is complex. Human physical growth in length and weight is generally characterized by rapid growth in early life, followed by a general deceleration in childhood and then a marked increase in late childhood associated with the onset of puberty (Grimm et al., 2011; Karlberg, 1987; Pan & Goldstein, 1998). Growth models have been used in various disciplines to understand and capture general features of growth processes. They have extensively been used in developmental research to understand biological as well as psychological processes at the individual or population level, using data collected longitudinally (Black & Krishnakumar, 1999; Botton et al., 2008; Ehrenkranz et al., 1999; Grimm et al., 2011; Nguyen et al., 2012; Olusanya & Renner, 2011; Skinner et al., 2004).

Modelling of such longitudinal growth data involves fitting a model that best describes the changes in the growth measurements of an individual or population over time (Goldstein et al., 2002; Pan & Goldstein, 1998). The fitted models can be used to summarize and interpolate the pattern of growth between measurement occasions and also identify critical periods in the growth process (Hauspie et al., 2004). Researchers have, thus, used growth models that can capture the non-linearity of the growth process.

Researchers have over the years developed and used several growth models. These can broadly be classified into two groups, namely structural (or parametric) and non-structural (non-parametric) models (Hauspie et al., 2004). Common structural models used include the Jenss-Bayley model, the Count model, Berkey-Reed 1st and 2nd order models, the Infant--Childhood--Puberty (ICP) model, the Preece-Baines model and the Gompertz, while most common non-structural models are polynomials and splines (Botton et al., 2008; Gasser & Molinari, 2004; Hauspie et al., 2004; Olusanya & Renner, 2011; Pan & Goldstein, 1998). The best model to describe the human growth process, be it at individual or population level, depends on the dimensions used (weight, height, skin-fold or circumferences), the frequency of the measurements (weekly, monthly, yearly) and the period of growth being investigated (infancy, childhood or adolescence) (Hauspie et al., 2004; Karlberg, 1987). Growth models that have fitted well to the infancy or childhood period include the Jenss-Bayley, the Berkey-Reed and the Count models (Hauspie et al., 2004). All of these models have functions that capture the rapid growth and then subsequent deceleration that takes place during this period of growth. The ICP model summarizes human growth into three overlapping components. The infancy component (birth to ∼3 years) is an extension of the foetal stage, is predominantly affected by maternal and nutritional factors. The childhood component is from 1 year to ∼11 years and is predominantly controlled by growth hormones. Simondon et al. (1992) used the first component of the ICP model to describe growth from birth to 13 months in Congolese infants.

Although non-structural models are easy to fit, they tend to be unstable at the extremities and do not define any particular form of the growth curve and, as such, their parameters do not have any biological interpretation (Hauspie et al., 2004; Singer & Willett, 2003).

There are several studies that have looked at child growth in low- and middle-income countries, but few have used longitudinal data, due to the limited number of longitudinal studies (Adair et al., 2009; Cameron et al., 1986; Fetuga et al., 2011; Guedes et al., 2010; Hauspie & Pagezy, 1989; Johnson et al., 2012b; Kalanda et al., 2005; Maleta et al., 2003b; Mushtaq et al., 2012; Olusanya & Renner, 2011; Pagezy & Hauspie, 1985; Simondon et al., 1992; Stein et al., 2010).

A number of studies have used the quadratic curve or some structural human growth models to model early child growth data. Table 1 shows a summary of some of these studies and the models used. Of these studies, only three compared several models to find one that best described the particular population. Furthermore, very few studies have used structural or non-structural models on African longitudinal growth data (Cameron et al., 1986; Olusanya & Renner, 2011; Pagezy & Hauspie, 1985; Simondon et al., 1992). Previous studies done in this setting have also not considered the whole of the childhood period from birth to age 10 years. Apart from differences in the period to which the models have been fitted, these studies fitted models to each individual child separately (Cameron et al., 1986; Hauspie & Pagezy, 1989; Pagezy & Hauspie, 1985; Simondon et al., 1992). This study aims to compare models that have previously been predominately used to model infant and early childhood growth such as the quadratic and Berkey-Reed model and those used in the late childhood period, such as the Jenss-Bayley and the adapted Jenss-Bayley models. This study aims to fit the models to the population growth data using mixed effects modelling. The rationale behind population-based growth modelling is that, while different individuals are quantitatively different, their growth over time has a similar shape. Thus, the objective of fitting a growth curve in this instance is to quantify this common shape, but at the same time take account of the between-individual differences in growth. As well as fitting individual curves, mixed effects modelling allows for fitting of a general population curve. The fixed part of a mixed model summarizes the mean structure (general population curve) and the random component of the model allows for variations in individual growth of the children. The other advantage of using mixed effects models is that they allow for modelling of longitudinal data which have a different number of measurement occasions or where some individuals have missing outcome measurements at some points or have unequal spaced intervals between measurements occasions. The importance of this flexibility in the analyses of longitudinal studies, where missing data are inevitable and where measurements on participants are more likely to be taken at the different times, can therefore not be emphasized. Mixed effects modelling also allows for inclusion of covariates that affect growth (Johnson et al., 2012b).

**Table 1.** Summary of some studies that have used structural and non-structural models to describe physical growth*.

	Reference	Study population	Model(s) used	Period of growth	Variable
1	Black and Krishnakumar (1999)	US (92% African-American)	Quadratic	0–6 years	Height, weight
2	Ehrenkranz et al. (1999)	US	Piece-wise quadratic	0–6 months	Weight
3	Martin-Gonzalez et al. (2012)	Spanish and Siberian	Kouchi	Birth–6 years	Height
4	Grimm et al. (2011)	US	Linear; Quadratic; Latent basis model, Preece-Baines	3–19 years	Height
5	Johnson et al. (2012b)	Indian	Berkey-Reed 1st order; Count; Quadratic	0–15 months	Weight
6	Botton et al. (2008)	French	Adapted Jenns-Bayley model (with a quadratic term)	0–12 years	Weight, height
7	Simondon et al. (1992)	Congolese (African)	Berkey-Reed 1st order; Count; Karlberg; Berkey-Reed 2nd order; Kouchi	0–13 months	Weight
8	Tilling et al. (2011)	Belarus	Fractional Polynomial	0–6.5 years	Weight, height
9	Flexeder et al. (2012)	German	Berkey-Reed 1st order	0–2 years	Weight, height
10	Steele (2008)	British	3rd Order Polynomial (cubic)	11–14 years	Height

* Publications found using Pubmed and Google-Scholar search. Search terms used: mathematical growth curve, child growth models, human growth model.

This study aims to compare four structural and two non-structural models that have been shown to fit well to the infant and childhood stage in high income country settings, by applying them to data from a South African (middle-income country) cohort. The objective of this study is to find a growth model that best describes physical growth of normal children from birth to 10 years in this setting using mixed effects modelling techniques.

## Subjects and methods

The study used weight and height measurements from 453 participants of the Bone-Health (BH) study as outcome variables. The BH Study is a sub-sample of the Birth-to-Twenty (Bt20) birth cohort set in Soweto-Johannesburg, South Africa. Of the 453 participants, 43 had a gestational age of less than 37 weeks (term) and were excluded from the analysis. The data comprised of anthropometric measurements at birth, 3 months, 6 months, 1 year, 2 years, 4 years, 5 years, 7/8 years, 9 years and at 10 years. All participants whose weight-for age *z*-scores (WAZ) or whose height-for-age were consistently (on at least three occasions) greater than +2 or less than −2 were excluded from analysis as these were considered outliers for growth within the context of the BH cohort population.

Only participants with at least five weight or height measurements were included in the study since the largest models have four parameters. Since height/length measurements were only taken from 3 months of age, there were two separate final ‘analysis data sets’ for modelling weight and height. The final ‘analysis data set’ for weight as outcome had 365 participants, while the one for height had 350 participants.

### Growth curve modelling

Before fitting the growth curve, descriptive statistics such as means, standard deviations and frequencies were calculated. *T*-tests were used to compare mean weights and heights at each measurement occasion. These comparisons were done on both the overall data set and the final ‘analysis data set’. Proportions of males, small for gestation age (SGAs) and firstborns in the overall data set were also compared to those in the final ‘analysis data set’ to see whether there were any differences in characteristics between the two datasets. Exclusion of children with less than five measurements did not affect the general population distribution by sex, parity or mean maternal age. Several growth models were fitted to the data using a mixed effects modelling approach. The sex of a participant was entered as a covariate to take into account known difference in growth between males and females. The study also explored any interaction between sex and age of a child. To be able to fit the growth models as linear models, other functions of the variable ‘age’ such as natural log of age, ln(age), and exponential of age were calculated.

The general structure of the models fitted is:

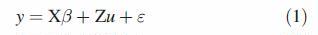

where *y* is the *n* × 1 vector of the observed weight/height, X is a *n* × *p* matrix of the fixed effects representing the different growth models (see Appendix 1), *β* is a *p* × 1 vector of the coefficients and Z is a *n* × *q* matrix of the random effects u. The *n* × 1 vector of errors is assumed to be multivariate normal with mean zero and variance of matrix 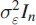.

The following four general model structures were defined from model (1) to test for the significance of the sex and age–sex interaction in the fixed effects and also the significance of different functions of age in the random component for each growth curve.

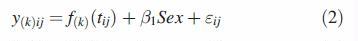


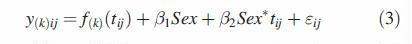


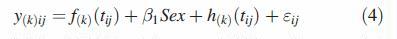


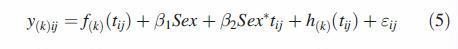

where 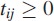 and represents the age of child *i* at measurement occasion *j*; *y_ij_* represents weight or height of child *i* at measurement occasion *j*; *ε_ij_* are random residuals; 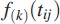 represents fixed effects; 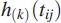 represents random effects; 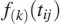 represents growth curve functions such as Berkey-Reed, Count and polynomial models; and 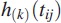 is a linear function with an intercept and slope (see Appendix 1). The random component allows for variations in the individual child’s starting measurement (intercept) and rate of growth (slope). For all of the weight models, the starting measurement is the child’s birth weight, while for models of height the starting measurement is the height at 3 months.

Models 2 and 3 are fixed effects models and were used to test the significance of adding the age–sex interaction to the growth curve function, 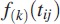. Models 4 and 5 are mixed effects models, with both fixed and random components, and were used to test for the effect of adding random effects into a model with sex and a model with an age–sex interaction, respectively. The two models were also used to test the significance of adding the age–sex interaction in a model with random effects. For models with random components, model building for the random components was done systematically by adding the intercept and slope (age) separately. The significance of each addition of terms was then tested using the likelihood ratio tests. The addition of higher order terms such as age^2^, ln(age) and age^3^ led to non-convergence of the models.

Comparisons of any nested models were done using likelihood ratio tests based on Maximum Likelihood Estimation (MLE). Variance components were also estimated using the MLE method. The unstructured variance–covariance option was used to estimate variance–covariance components. This option allows estimates to be distinct, unlike the independent or identity options that confine the variance–covariance patterns to particular values or patterns. Since the variance components describe the variability in the individual growth of the children, taking into account the initial birth weight, which also varies, the unstructured variance–covariance is the most appropriate option to use when modelling infant and child growth.

The Akaike Information criterion (AIC) and Bayesian Information Criterion (BIC) were used to compare non-nested models (Jones, 2011; Singer & Willett, 2003). Models with lower values of the AIC and BIC are considered better fitting. The median and maximum values of absolute residuals were used to determine models that were fitting well to the data over time (Royston & Altman, 1997). Smaller median and maximum values of the absolute residuals are also indicative of a better fitting model. Ranks were used to compare AIC, BIC, median and maximum values of absolute residuals. The median and maximum absolute residuals were also ranked at each measurement occasion, as an indicator of how well the model is fitting at each point relative to the other models. The overall ranks for all the goodness of fit statistics were then compared using the Kruskal--Wallis test. The Kruskal--Wallis test was also performed on the actual median and maximum values of the absolute residuals. Residual analysis was used to check for normality using normal probability plots. Most of the statistical analyses were done using Stata Version 11, except for fitting of the Jenss-Bayley and the Adapted Jenss-Bayley models, which was done using the Proc nlmixed procedure in SAS 9.3. All tests were conducted at the 5% significance level.

**Table 2.** Goodness-of-fit statistics from fitting growth models to weight measurements (in kg) from birth to 10 years.

		Models fitted					
		Berkey-Reed 1	Count	Jenss-Bayley	Adapted Jenss-Bayley	2nd Order Polynomial	3rd Order Polynomial
Variance components^a^	Random Intercept	0.160 (0.072, 0.355)	0.117 (0.035,0.389)	0.008 (−0.173, 0.188)	0.008 (−0.17, 0.181)	0.005 (0.0001, 0.166)	0.249 (0.142, 0.436)
	Random Slope	0.0013 (0.001, 0.002)	0.0013 (0.001, 0.002)	0.0013 (0.001, 0.002)	0.0013 (0.001, 0.0014)	0.001 (0.0010, 0.002)	0.0013 (0.001. 0.002)
	Covariance	−0.002 (−0.006, 0.001)	−0.003 (−0.007, 0.001)	−0.002 (−0.006, 0.003)	−0.002 (−0.006, 0.003)	−0.002 (−0.007, 0.002)	−0.005 (−0.01, −0.0006)
	Random Residuals	1.98 (1.86, 2.10)	2.35 (2.02, 2.50)	3.06 (2.87, 3.25)	3.05 (2.87, 3.23)	3.051 (2.87, 3.24)	1.95 (1.83, 2.07)
Information criterion	AIC	10 758	11 127	11 704	11 693	11 684	10 758
	BIC	10 811	11 175	11 739	11 724	11 708	10 811
Absolute residuals^b^,^c^	Median	0.62 (0.28, 1.20)	0.72 (0.33, 1.35)	0.99 (0.44, 1.89)	0.99 (0.44, 1.84)	0.99 (0.44, 1.84)	0.86 (0.42, 1.37)
	Maximum	8.46	8.80	8.53	8.66	8.66	7.16
Median absolute residuals^c,d^	Birth (*n* = 365)	0.32 (0.15, 0.54)	0.54 (0.27, 0.83)	2.03 (1.70, 2.37)	1.90 (1.59, 2.26)	1.91 (1.59, 2.25)	0.99 (0.67, 1.30)
	3 m (*n* = 126)	1.14 (0.76, 1.57)	0.61 (0.30, 1.05)	0.58 (0.31, 0.90)	0.58 (0.34, 0.99)	0.58 (0.34, 0.99)	0.72 (0.47, 1.14)
	6 m (*n* = 87)	0.55 (0.31, 0.89)	1.18 (0.55, 1.66)	1.34 (0.77, 1.96)	1.41 (0.84, 2.03)	1.42 (0.84, 2.03)	1.47 (0.82, 1.82)
	Year 1 (*n* = 270)	0.85 (0.34, 1.44)	1.19 (0.55, 1.82)	1.74 (1.00, 2.53)	1.77 (1.02, 2.55)	1.77 (1.02, 2.56)	1.20 (0.55, 1.72)
	Year 2(*n* = 264)	0.79 (0.38, 1.43)	0.82 (0.39, 1.41)	1.16 (0.52, 1.94)	1.13 (0.51, 1.90)	1.13 (0.51, 1.90)	0.77 (0.38, 1.22)
	Year 4 (*n* = 345)	0.65 (0.32, 1.18)	0.76 (0.32, 1.30)	0.74 (0.35, 1.34)	0.73 (0.35, 1.31)	0.73 (0.35, 1.31)	0.76 (0.35, 1.33)
	Year 5 (*n* = 306)	0.63 (0.25, 1.09)	0.73 (0.34, 1.26)	0.60 (0.25, 1.12)	0.66 (0.25, 1.11)	0.66 (0.25, 1.11)	0.59 (0.30, 1.04)
	Year 7/8 (*n* = 322)	0.76 (0.35, 1.74)	0.83 (0.39, 1.83)	0.87 (0.40, 1.85)	0.92 (0.44, 1.89)	0.92 (0.44, 1.89)	0.93 (0.48, 1.41)
	Year 9 (*n* = 299)	0.48 (0.23, 0.89)	0.45 (0.23, 0.87)	0.63 (0.29, 0.99)	0.60 (0.29, 0.97)	0.60 (0.29, 0.97)	0.51 (0.23, 0.90)
	Year 10 (*n* = 313)	0.79 (0.32, 1.68)	0.96 (0.34, 1.94)	0.71 (0.35, 1.51)	0.76 (0.35, 1.61)	0.76 (0.35, 1.61)	1.06 (0.55, 1.57)
Maximum absolute residuals^d^	Birth (*n* = 365)	1.27	1.89	3.44	3.32	3.32	2.32
	3 m (*n* = 126)	3.54	2.62	2.60	2.70	2.71	2.61
	6 m (*n* = 87)	2.60	3.60	3.94	4.03	4.02	3.75
	Year 1 (*n* = 270)	5.57	6.25	6.97	7.01	7.01	6.00
	Year 2 (*n* = 264)	4.14	4.03	4.99	4.96	4.95	3.51
	Year 4 (*n* = 345)	6.33	6.77	6.10	6.24	6.24	6.85
	Year 5 (*n* = 306)	4.37	4.81	4.28	4.45	4.45	3.81
	Year 7/8 (*n* = 322)	7.31	7.40	7.25	7.32	7.32	6.18
	Year 9 (*n* = 299)	5.05	5.31	5.18	5.24	5.24	4.78
	Year 10 (*n* = 313)	8.45	8.80	8.53	8.66	8.66	7.16
Sum of the rank		56	89.5	96	109	107	68.5
Kruskal--Wallis Rank Sum	*p* < 0.001	1076	1956	2096	2423	2361	1412
% positive raw residuals	Birth (*n* = 365)	174 (48)	45 (12)	0 (0)	0 (0)	0 (0)	3 (1)
	3 m (*n* = 126)	6 (5)	96 (76)	79 (63)	89 (71)	89 (71)	110 (87)
	6 m (*n* = 87)	51 (59)	79 (91)	81 (93)	82 (94)	82 (94)	85 (98)
	Year 1 (*n* = 270)	215 (80)	241 (89)	252 (93)	257 (95)	257 (95)	243 (90)
	Year 2 (*n* = 264)	166 (63)	149 (56)	214 (81)	212 (80)	212 (80)	118 (45)
	Year 4 (*n* = 345)	160 (46)	106 (31)	203 (59)	182 (53)	182 (53)	97 (28)
	Year 5 (*n* = 306)	139 (45)	78 (25)	163 (53)	139 (45)	139 (45)	182 (59)
	Year 7/8 (*n* = 322)	50 (16)	41 (13)	42 (13)	35 (11)	35 (11)	211 (66)
	Year 9 (*n* = 299)	115 (38)	131 (44)	88 (29)	96 (32)	96 (32)	180 (60)
	Year 10 (*n* = 313)	226 (72)	264 (84)	195 (62)	205 (65)	205 (65)	103 (33)

^a^Variance components are given with their 95% CI. ^b^Distribution of absolute residuals for each model. ^c^Medians are given with their Interquartile Range (IQR). ^d^Distribution of absolute residuals for each model at each measurement occasion. AIC, Akaike Information Criterion; BIC, Bayesian Information Criterion.

## Results

### Descriptive statistics

Of the 365 participants used in modelling weight, 190 (52%) were males, 139 (44%) were first born and 25 (6.9%) had small birth weight for their gestation age (SGA), and the mean age of the mother was 25.1 years (SD = 6.1).

Comparisons of the mean weight and height measurements by sex or birth weight [SGA vs appropriate for gestation age (AGA)] were made at each measurement occasion (results not shown). There were slight differences in average weight and height from birth to ∼2 years between AGA and SGA infants, indicating smaller babies gaining weight and height faster (0.05 < *p* < 0.10).

In line with biological expectations, there was a significant difference in average weight and height between males and females at most of the measurement occasions, especially during the early years, with boys weighing on average more than girls and also being taller than girls. There were significant differences in average weight between boys and girls from birth to 1 year. Similar trends were observed in mean height between the two sexes from 3 months to ∼2 years, with boys being on average taller than girls. There were no significant differences in mean weight or height between boys and girls from 2 years to ∼9 years. At 10 years, the girls were on average heavier than boys, although the difference was not significant.

**Figure 1.**
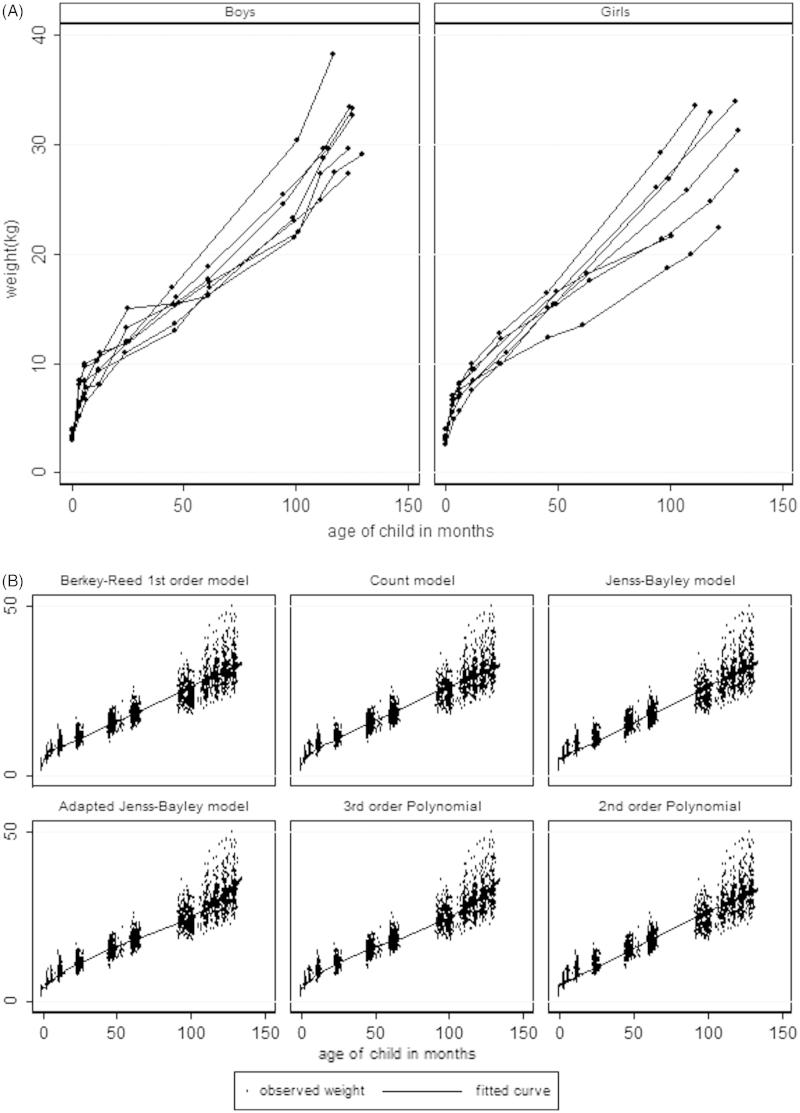
Graphs of weight profiles and growth models fitted to weight from birth to 10 years.

**Figure 2.**
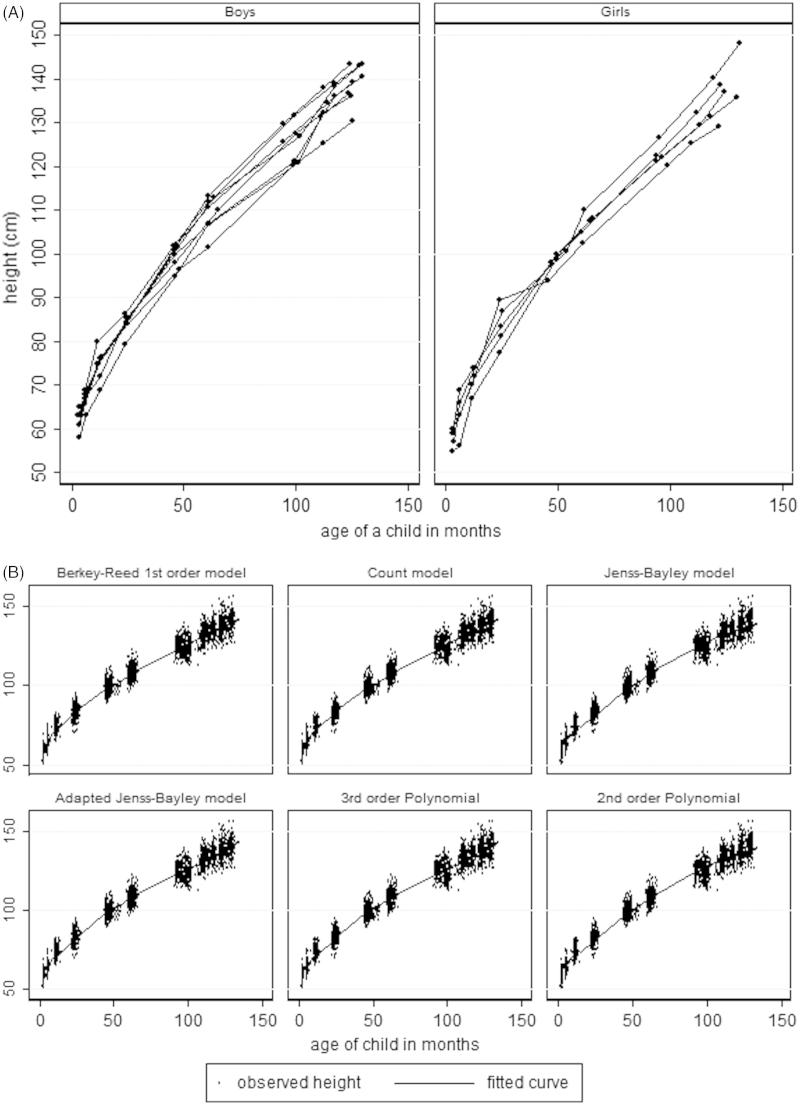
Graphs of height profiles and growth models fitted to height from 3 months to 10 years.

Weight and height profiles for a random sample of boys and girls have been shown in the first graphs of Figures 1 and 2. The weight profiles show some rapid weight gain in the first year of life. A similar trend is shown by the height profiles.

### Fitted growth models for weight of children

The parametric growth curve functions used were the Berkey-Reed 1st and 2nd order model, the Count model, Jenss-Bailey model and the adapted Jenss-Bayley model. The non-structural models fitted were the 2nd and 3rd order polynomials. The Berkey-Reed 2nd order model was highly correlated with its 1st order model. Thus, the 1st order which has fewer parameters was used in the modelling, considering that the number of measurement occasions per child was also small. There were significance effects of adding the random intercepts to models with ‘sex–age interactions’ for all growth functions (all *p* values <0.05). Only age was included in the random component of all of the models, since the addition of higher order functions of age led to non-convergence of the models.

The graphical representations of the fitted curves on the observed weight are shown in Figure 1. The Berkey-Reed 1st order model had the best fit at all of the measurement occasions, with the curves passing almost at the middle of the observed measurements at each time point (except at 3 months). The Count and the 3rd order Polynomial models also fitted well at most of the measurement occasions. All of the models except for the Berkey-Reed 1st order model do not fit well to the first four measurement occasions (birth to 1 year). The 3rd order Polynomial picks up the rapid weight gain from ∼9 years, while the other four models are approximately linear and do not allow for this weight gain.

The findings from the graphical representation were also supported by the percentage of positive raw residuals at each measurement occasion (Table 2), with close to 50% positive residuals at each measurement occasion being an indication of a good fitting model. From Table 2, the quadratic, the adapted Jenss-Bayley and Jenss-Bayley models had no positive residuals at birth, implying that all predicted birth weights were higher than the observed birth weights. Apart from the Berkey-Reed model, the other models had a poor fit at birth. At year 1, all six models did not fit well, with more than 80% of the residuals being positive. In general the Jenss-Bayley and quadratic models had a poor fit from birth to 2 years but fitted better in later years, while the 3rd order Polynomial, the Count and the adapted Jenss-Bayley did not fit well up to ∼1 year. Although the percentages of positive residuals from fitting a Berkey-Reed model were consistently close to 50% at most of the measurement occasions, the model fitted poorly at 3 months and at 7/8 years. At 3 months, only 6% of predicted weights were less than the observed weight, while at 7/8 years, 80% of the predicted weights were less than the observed. This was also shown by the large median and maximum absolute residuals.

Both the adapted Jenss-Bayley and the 3rd order polynomial also did not fit well from birth to ∼1 year, but fitted better in the later years. Based on the overall trend in percentage of positive residuals, it can be concluded that the Berkey-Reed model fits better than the other five models.

The random intercept (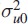) represents the variation in the initial value. For models fitted from birth, the initial value represents the birth weight of a child. The random intercept allows for estimation of an individual child’s birth weight, thus the model does not constrain individuals to have the same birth weight. The random slope (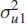) in the models allows for the estimation of differences in individual growth trajectories, linear in age. The results in Table 2 show that the variances (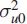) for the random intercept ranged from 0.001–0.245, with the 3rd order Polynomial model having the largest variance estimate and the Jenss-Bayley having the largest standard error of the estimate. However, the confidence intervals for 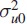 for all of the models overlapped, indicating that there were no significant differences in the random intercepts of the six fitted growth curves. All models had similar estimates of variance (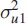 = 0.001) of the random slope and similar standard errors. The estimates for the covariance (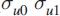) of the intercept and slope in all of the six models were all negative. Although the covariance estimates for all the models are also negative, most of the confidence intervals included zero, indicating a non-significant negative covariance. Only the 3rd order polynomial model had a significant negative covariance. A significant negative covariance indicates that those with low initial values (low birth weight) grow faster than those with higher initial values (normal/large babies).

The estimates for the effects of sex differences on weight ranged from −0.44 to −0.53, showing that girls were on average about half a kilogram lighter than boys. The 95% confidence limits ranged from −0.75 to −0.26, indicating that differences in weight between weight of boys and girls range from ∼300–800 g. The confidence intervals for the effect of sex in all of the models overlapped, again indicating that there is no significant difference in the estimation of the effect by the six models (Appendix 2). The effect of age and sex interaction was significant in all six models. The estimate for this effect for all six models was 0.01, indicating an average monthly increase of 10 g in girls relative to boys. All terms which are a function of age of the participant in all the models were highly significant (*p* < 0.001). This shows the importance of applying the different functions of age to appropriately model the shape of a growth curve.

### Goodness of fit tests for models on weight

The Akaike Information Criterion (AIC), Bayesian Information Criterion (BIC), median and maximum values of absolute residuals and the variance (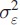) of residuals were used to assess the goodness-of-fit of all of the models (Table 2). For all the goodness-of-fit statistics, the smaller the value of the statistics, the better the model is fitting to the data.

Both the Berkey-Reed and the 3rd order Polynomial models had the smallest AIC and BIC values (10758 and 10811, respectively) and the Berkey-Reed had the smallest overall median absolute residual of 0.62, with an interquartile range of 0.28–1.20. It also had consistently the smallest median of the absolute residuals at most of the 10 measurement occasions. The maximum values for the absolute residuals for the six models range from 7.16–8.80, with the 3rd order Polynomial model having the smallest maximum value.

The ranks of the AIC, BIC and the median and maximum absolute residual values show the Berkey-Reed having the smallest sum of the ranks, with 17 out of 25 ranks for the model being less than 3. The Kruskal--Wallis test on the ranks of the goodness-of-fit statistics showed significant differences in the ranks (*p* < 0.001) and the Berkey-Reed model had the smallest rank sum, followed by the 3rd order polynomial model.

Although there were no significant differences in the values of the median absolute residuals (*p* = 0.59), the Berkey-Reed model had the smallest sum of the ranks, indicating that the model had consistently smaller median absolute residuals at all of the measurement occasions. Similarly there were no significant differences in the sum of the ranks of the maximum absolute residual values amongst the models (*p* = 0.92), but the Berkey-Reed model had the smallest rank sum, again indicating consistently smaller values of absolute residuals for this model.

The estimates of the variance (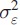) of residuals after fitting the models to the data ranged from 1.95–3.06, with the 3rd order Polynomial model having the smallest value and the Jenss-Bayley model the largest value.

### Fitted growth models for height of children

Figure 2 shows the graphical representation of the six models for height fitted from 3 months to 10 years, showing the Count, the adapted Jenss-Bayley and Berkey-Reed 1st order models fitting well to the data at almost all the measurement occasions. Table 3 also shows the percentage of positive residuals from fitting the six models. The percentage of positive residuals from fitting the adapted Jenss-Bayley or Berkey-Reed 1st order model is close to 50% at almost all the measurement occasions. All of the models, except the 3rd order Polynomial, did not fit well at year 2. They either over-estimated (small percentage of positive residuals) or under-estimated (large percentage of positive residuals). The adapted Jenss-Bayley also did not fit well at year 7/8, while the Jenss-Bayley model did not fit well at years 1 and 5. The 2nd order polynomial did not fit well at almost all points except at age 5 and 9 years. The 2nd and 3rd order polynomial models had very low percentages of positive residuals at 3 months and a high percentage from year 1 to year 4, indicating over-estimation at 3 months and under-estimation from year 1 to year 4.

**Table 3.** Goodness-of-fit statistics from fitting growth models to height measurements (in centimetres) from 3 months to 10 years.

		Models fitted					
		Berkey-Reed 1	Count	Jenss-Bayley	Adapted Jenss-Bayley	2nd Order Polynomial	3rd Order Polynomial
Variance components^a^	Random Intercept	6.32 (5.14, 7.78)	6.25 (5.08, 7.70)	6.45 (5.10, 7.80)	6.21 (4.91, 7.51)	6.78 (5.40, 8.52)	6.65 (5.39, 8.20)
	Random Slope	0.002 (0.001, 0.0021)	0.002 (0.001, 0.0021)	0.002 (0.001, 0.0022)	0.002 (0.001, 0.0022)	0.002 (0.001, 0.0022)	0.002 (0.001, 0.0021)
	Covariance	0.007 (−0.008, 0.022)	0.007 (−0.007, 0.022)	0.007 (−0.008, 0.022)	0.007 (−0.007, 0.023)	0.005 (−0.011, 0.022)	0.006 (−0.009, 0.022)
	Random Residuals	4.30 (4.01, 4.60)	4.33 (4.04, 4.64)	4.48 (4.17, 4.78)	4.38 (4.07, 4.68)	6.53 (6.10, 7.00)	4.75 (4.44, 5.09)
Information criterion	AIC	11510	11519	11586	11539	12259	11696
	BIC	11562	11565	11620	11577	12305	11747
Absolute residuals^b^,^c^	Median	0.94 (0.41, 1.75)	0.97 (0.43, 1.80)	0.95 (0.42, 1.80)	0.90 (0.40, 1.69)	1.11 (0.51, 2.09)	1.00 (0.44, 1.86)
	Maximum	12.87	12.95	12.64	12.46	11.16	11.76
Median absolute residuals^c^,^d^	3 m (*n* = 119)	1.37 (0.48, 2.37)	1.38 (0.64, 2.37)	1.24 (0.66, 2.50)	1.39 (0.52, 2.36)	3.58 (2.37, 5.20)	1.86 (1.07, 3.44)
	6 m (*n* = 87)	1.31 (0.68, 2.29)	1.23 (0.51, 2.40)	1.34 (0.78, 2.25)	1.20 (0.52, 2.59)	1.29 (0.57, 2.92)	1.24 (0.56, 2.33)
	Year 1 (*n* = 259)	1.18 (0.57, 1.95)	1.17 (0.58, 1.90)	1.54 (0.68, 2.40)	1.09 (0.58, 2.01)	1.65 (0.76, 2.81)	1.79 (0.85, 2.83)
	Year 2 (*n* = 303)	1.32 (0.59, 2.19)	1.34 (0.62, 2.21)	1.19 (0.54, 2.12)	1.08 (0.51, 1.96)	1.22 (0.55, 2.05)	0.95 (0.38, 1.75)
	Year 4 (*n* = 336)	0.94 (0.43, 1.59)	0.93 (0.44, 1.64)	0.90 (0.40, 1.58)	0.96 (0.47, 1.78)	1.06 (0.53, 1.80)	0.91 (0.43, 1.66)
	Year 5 (*n* = 303)	1.09 (0.45, 1.77)	1.17 (0.51, 1.91)	0.98 (0.43, 1.62)	0.96 (0.44, 1.67)	0.83 (0.37, 1.47)	0.80 (0.39, 1.42)
	Year 7/8 (*n* = 314)	0.84 (0.42, 2.14)	0.86 (0.42, 2.13)	0.96 (0.41, 2.20)	0.88 (0.36, 2.33)	1.48 (0.71, 2.86)	1.08 (0.43, 2.37)
	Year 9 (*n* = 299)	0.52 (0.25, 1.03)	0.53 (0.25, 1.06)	0.54 (0.24,1.06)	0.52 (0.21, 0.99)	0.57 (0.25, 1.01)	0.71 (0.37, 1.32)
	Year 10 (*n* = 308)	0.81 (0.31, 1.39)	0.84 (0.33, 1.37)	0.82 (0.31,1.38)	0.77 (0.42, 1.35)	1.10 (0.52, 1.85)	0.92 (0.36, 1.58)
Maximum absolute residuals^d^	3 m (*n* = 119)	6.98	6.53				
	6 m (*n* = 87)	7.46	7.63	7.30	7.83	8.65	7.70
	Year 1 (*n* = 259)	5.32	5.05	6.15	4.96	6.89	6.71
	Year 2 (*n* = 303)	12.87	12.95	12.64	12.46	10.44	11.76
	Year 4 (*n* = 336)	5.80	5.67	6.01	5.71	6.24	6.58
	Year 5 (*n* = 303)	10.12	10.27	9.88	9.99	9.16	9.17
	Year 7/8 (*n* = 314)	6.78	6.73	6.64	7.47	8.27	6.33
	Year 9 (*n* = 299)	4.76	4.78	4.69	4.88	4.83	4.32
	Year 10 (*n* = 308)	9.55	9.47	9.45	10.26	11.09	8.89
Sum of the Ranks		70	75	76.5	68	111	84.5
Kruskal--Wallis Rank Sum	*p* = 0.002	1348	1463	1496	1300	2297	1686
% positive raw residuals	3 m (*n* = 119)	66 (55)	74 (62)	37 (31)	69 (58)	1 (1)	16 (13)
	6 m (*n* = 87)	49 (56)	45 (52)	50 (57)	43 (49)	26 (30)	45 (52)
	Year 1 (*n* = 259)	168 (65)	150 (58)	202 (78)	143 (55)	202 (78)	215 (83)
	Year 2 (*n* = 303)	62 (20)	62 (20)	66 (22)	96 (32)	218 (72)	134 (44)
	Year 4 (*n* = 336)	186 (55)	199 (59)	154 (46)	224 (67)	230 (68)	126 (38)
	Year 5 (*n* = 303)	234 (77)	248 (82)	222 (73)	222 (73)	162 (53)	167 (55)
	Year 7/8 (*n* = 314)	152 (48)	154 (49)	165 (52)	86 (27)	29 (9)	181 (58)
	Year 9 (*n* = 299)	189 (63)	184 (62)	202 (67)	156 (52)	141 (47)	237 (79)
	Year 10 (*n* = 308)	137 (44)	125 (41)	133 (43)	188 (61)	245 (80)	106 (34)

^a^Variance components are given with their 95% CI. ^b^Distribution of absolute residuals for each model. ^c^Medians are given with their Interquartile Range (IQR). ^d^Distribution of absolute residuals for each model at each measurement occasion. AIC, Akaike Information Criterion; BIC, Bayesian Information Criterion.

The estimates for variances (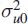) of the random intercepts (variation in height at 3 months) ranged from 6.25–6.82, with the Count model having the smallest estimate. However, the confidence intervals for estimates of the random intercept (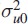)for all the models overlapped, indicating that there were no significant differences in the estimation of random intercept between the different shapes of curves fitted across the models. The estimate of the covariance (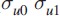) of the random intercept and slope for all the models were all positive and not significantly different from each other (confidence intervals overlapped). All of the confidence intervals for the covariance estimates included zero, indicating independence between one’s initial height at 3 months (random intercept) and their growth rates (random slope). All of the models had similar estimates of variance (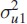) of the random slope, with overlapping confidence intervals.

The estimates for the effects of sex differences on height ranged from −1.67 to −1.49, showing that girls were on average ∼1.5–2.0 centimetres shorter than boys. The confidence intervals for the effect of sex in all the models overlapped, indicating that there were no significant differences in the estimates from the different models. The sex–age interaction estimate for all the models was 0.02, indicating an average monthly increase in girls’ height of ∼0.2 mm relative to boys. From ∼4 years to 7/8 years, there were no differences in the average height between boys and girls. As with weight models, all terms which are a function of age of the participant in all the models were highly significant (all *p* < 0.001), indicating the relationship between physical growth and one’s age.

### Goodness-of-fit tests for models on height

The AIC values ranged from 11 510–12 259, with the Berkey-Reed model having the smallest AIC and BIC values as well as the smallest estimates for the random residuals (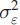). The Count model also had smaller AIC and BIC values compared to the other four models. The overall median absolute residuals ranged from 0.90–1.11, with the adapted Jenss-Bayley having the smallest overall median of absolute residuals and the quadratic model having the largest value. All models did not fit well to the data at year 2, producing very large maximum absolute residuals. This is could be due to the wide variation in height measurements at this data collection waves. The height measurements ranged from 70–95 cm for age that ranged from 22.5–28 months. The wide variation in the measurements could have been due to the changes in measurement procedure from sitting to standing positions. The Kruskal--Wallis test on all the ranks of the goodness-of-fit statistics showed significant differences in ranks, with the adapted Jenss-Bayley model having the smallest rank sum.

At each measurement occasion, the adapted Jenss-Bayley and the Berkey-Reed models have consistently smaller maximum and median values of absolute residuals. However, the Kruskal--Wallis test on the maximum and median absolute values showed no significant differences (*p* values of 0.57 and 0.72, respectively). The adapted Jenss-Bayley had the smallest sum of ranks followed by the Berkey-Reed model.

## Discussion

The paper has used mixed effects models to compare the fitness of different infancy and childhood growth models and has demonstrated the benefits of using mixed effects modelling to understand the general patterns of growth in children. Most previous studies in Low and Middle Income Countries (LMIC) have used growth centiles to model growth, with an aim to monitor growth and detect timing of growth faltering due to malnutrition by comparing child growth to set growth reference charts (Fetuga et al., 2011; Johnson et al., 2012b; Maleta et al., 2003a; Mushtaq et al., 2012; Nguyen et al., 2012; Kalanda et al., 2005; Stein et al., 2010). Of the studies from LMIC that used growth models, none modelled growth beyond 2 years of age and none of them except for the study by Johnson et al. (2012b) used mixed effects modelling to fit the growth models (Olusanya & Renner, 2011; Pagezy & Hauspie, 1985; Simondon et al., 1992). Mixed effects modelling of physical growth measurements allows for the estimation of general population growth pattern as well as that of an individual child and allows for the incorporation of other factors that can affect child growth in the modelling process (Johnson et al., 2013). Before the advent of mixed effects models, growth curves had to be fitted to each individual child separately (Cameron et al., 1982). Unlike other methods for analysis of longitudinal data such as generalized estimating equations (GEE) and multivariate analysis of variance (MANOVA), mixed effects modelling allows for differences in timings and number of data points per individual (Twisk & de Vente, 2002; Twisk, 2004).

Furthermore, covariance estimates in a mixed effects growth model explain the relationship between starting values and growth trend. There was evidence in the study of SGA exhibiting rapid growth in infancy, as shown by the negative covariance estimates. Negative covariance estimates indicate that those with low initial values (e.g. low birth weight) grow faster than those with higher initial values (normal/large babies), while positive covariance indicates that those with initial values below the mean are likely to remain below the mean and those with initial values above the mean maintain that status (Singer & Willett, 2003; Zimmerman & Nunez-Anton, 2001). Johnson et al. (2012a, b), using mixed effects modelling to fit the Berkey-Reed model, also found negative covariance estimates in both Indian and British populations. Although this study did not show as strong evidence of catch-up growth as the earlier studies by Johnson et al., this could be due to the fact that we are looking at growth from birth to 10 years, whereas the earlier studies focused on the first 2 years of life when it would be expected that the effect of catch-up growth would be strongest.

In this study, the non-convergence of the models after addition of higher order term could have been due two factors; (1) the limited number of measurement occasions, with long and unequally spaced time intervals, and (2) the lack of variation that is seen in the deceleration of growth across individuals in the early childhood period. Steele (2008), also using mixed effects modelling, showed a significant effect in adding the quadratic term (age^2^) to the random component of a 3rd order polynomial model. However, the data used by Steele had nine equally spaced measurement occasions, between the ages of 11–14 years (during puberty when individual variation in acceleration and deceleration of growth occurs), while the maximum number of data points in this study is seven spread over a 10-year period. Since the addition of the quadratic or ln(age) term in the random component would allow for variations in the period of deceleration in growth amongst the children, the few measurement occasions over a wide age range might have led to computational problems, in that the shape of the growth curve is different from the one being imposed by the model (Simondon et al., 1992). The growth velocity curves (not shown) for the six models showed a similar period of deceleration in growth. This could be the reason why allowing for variation in deceleration led to computational problems with this data set and why the results of this study are different to earlier studies.

Even though most of the studies in LMIC that have used the Berkey-Reed 1st order model have applied it to infant growth data (0–2 years), our study found that it fitted well to the childhood period, compared to the other five models (Hauspie & Pagezy, 1989; Johnson et al., 2012b; Pagezy & Hauspie, 1985; Simondon et al., 1992). In a study of Indian children, Johnson et al. (2012b) found that the Berkey-Reed 1st order model fitted better to infant weight and height data compared to other models such as the Count and 2nd order polynomial (quadratic) models. Studies that have modelled weight or height beyond 2 years have used models such as the Jenss-Bayley, Kouchi, adapted Jenss-Bayley and quadratic models and none of these did a comparative study on the fitness of the different models (Black & Krishnakumar, 1999; Botton et al., 2008; Dwyer et al., 1983; Martin-Gonzalez et al., 2012; van Dommelen et al., 2005). Some studies have used the quadratic model mainly for its simplicity and not necessarily because the model fits well to the data (Ehrenkranz et al., 1999; Grimm et al., 2011). Biologically, the quadratic model would not be appropriate for the age period under study, as it would not be able to capture the possible acceleration in growth that takes place pre-puberty. Quadratic models have been found to be inappropriate in capturing growth characteristics over longer time intervals (Hauspie et al., 2004).

Although our study found that the Jenss-Bayley model did not fit well in the first year of life, this could be due to the limited number of measurement occasions, leading to the failure by the model to capture the asymptotic nature of the curve in infancy. Further, the limited number of individuals with weight at 3 and 6 months could also have been attributed to the failure for the model to fit well at these points. Although the adapted Jenss-Bayley model in general fitted better than the Jenss-Bayley, it also did not fit well in the first year of life. The quadratic term added to the Jenss-Bayley model by Botton et al. (2008) introduced some deceleration effect to minimize the effect of the exponential term (rapid growth) and this possibly helped in capturing the growth in infancy better than if there is just a linear term. It is worth noting that the study by Botton et al. (2008) did not compare the goodness of fit of the adapted Jenss-Bayley model with any of the models used in this study. They validated their residual analysis using piece-wise models.

In general, all of the models seemed to fit to height data better than the weight data, as was evidenced by the non-significant differences in the median values of the absolute residuals. One of the challenges in modelling weight as opposed to height is that individual weight can fluctuate and is more sensitive to changes in ecological and environmental factors such as nutrition, while height is monotonic (i.e. increases with age) (Dwyer et al., 1983). Human growth models are monotonic functions, primarily derived to model monotonic biological processes. Thus, ecological and environmental influences that vary those monotonic functions are likely to lead to poorer fitting models, depending on the amount of variation that is driven by biological processes and the amount driven by ecological and environmental influences. Despite this, several studies have shown that the models can fit equally well to weight measurements (Botton et al., 2008; Dwyer et al., 1983; Johnson et al., 2012a, b; Pagezy & Hauspie, 1985; Simondon et al., 1992; van Dommelen et al., 2005).

### Limitations

The main limitations to this study are the limited data, especially during the first 24 post-natal months due to missing data on the growth measurement variables and the number of data collection waves. Having more participants with growth measurements at 3 months and 6 months or more data collection waves (monthly collection) may have helped in improving the fit of the different models to the data and in picking up the rapid growth in infancy more precisely.

Another factor that could have affected the fit of the models is the time period (birth to 10 years), which might have included the pubertal take-off period, as a study by Jones et al. (2009) showed that the average age at onset of puberty in this population is ∼10 years. However, excluding the measurements at year 10 would have led to a further reduction in the sample size.

Although the number of individuals with a minimum of five weight or height measurements was relatively small due to missing data, the distribution of weight and height amongst these individuals with data was not different from that of the other height and weight measurements taken in the cohort.

## Conclusion

Based on AIC and BIC values and also the median and maximum of absolute residuals, the best growth model when modelling weight during infancy and childhood (up to 10 years) in this South African context, has been shown to be the Berkey-Reed 1st order model. The Count and the 3rd order Polynomial are also good, as they pick up the rapid growth in infancy, the slowing down in childhood and then the accelerated growth at the beginning of puberty (∼9 years). The other advantage of the Count model is that it has one parameter less than the Berkey-Reed or the 3rd order Polynomial, meaning that fewer data points are required to fit the model. The Adapted Jenss-Bayley model fitted height measurements better than the other models. Also found to fit height data well were the Berkey-Reed 1st order and the Count models. Overall, the simpler linear Berkey-Reed model seems to fit well to both height and weight for the period from birth to pre-puberty. Simondon et al. (1992) found the Berkey-Reed model fitted best to African infant growth data. This study extended the findings of Simondon et al. to confirm that the model continues to fit well into late childhood (up to 10 years), even though it did not fit well to weight at 3 months, possibly due to limited data at this point, and at 7/8 years due to failure to capture the pre-pubertal growth spurt. A study with shorter intervals between data collection waves in the first 24 months of life would also help in improving the accuracy in fitting the models, since children undergo rapid growth during this period. This study has also demonstrated how mixed effects modelling can be used to compare the fitness of different infancy and childhood growth models.

## Declaration of interest

The authors report no conflicts of interest. The authors alone are responsible for the content and writing of the paper.

## Supplementary Material

Supplementary Material

## Notes

Supplementary material available online
