# Supplementary material for: Multi-level modelling of longitudinal child growth data from the Birth-to-Twenty Cohort: a comparison of growth models
Source: Ann Hum Biol. 2013 Oct 11;41(2):168–79. doi: 10.3109/03014460.2013.839742 (PMC4219852; doi:10.3109/03014460.2013.839742)
Supplement: Supplementary Material [file iahb_a_839742_sm0001.pdf]

Appendix 1: Growth models fitted to weight and height

(1) The Berkey-Reeds 1st order model:

$$y_{ij} = \beta_0 + \beta_1 t_{ij} + \beta_2 \ln(t_{ij}) + \beta_3 \frac{1}{t_{ij}} + \varepsilon_{ij} \quad j = 1,2,....k\ ,\ i = 1,2....n$$

(2) The Count model:

$$y_{ij} = \beta_0 + \beta_1 t_{ij} + \beta_2 \ln(t_{ij} + 1) + \varepsilon_{ij} \quad j = 1,2,....k\ ;\ i = 1,2....n$$

(3) The Jenss-Bayley model:

$$y_{ij} = \beta_0 + \beta_1 t_{ij} - \exp(\beta_2 + \beta_3 t_{ij}) + \varepsilon_{ij} \quad j = 1,2,....k\ ;\ i = 1,2....n$$

(4) The adapted Jenss-Bayley model

$$y_{ij} = \beta_0 + \beta_1 t_{ij} + \beta_2 t_{ij}^2 - \exp(\beta_3 + \beta_4 t_{ij}) + \varepsilon_{ij} \quad j = 1,2,....k\ ;\ i = 1,2....n$$

(5) The 2nd Order Polynomial:

$$y_{ij} = \beta_0 + \beta_1 t_{ij} + \beta_2 t_{ij}^2 + \varepsilon_{ij} \quad j = 1,2,....k\ ;\ i = 1,2....n$$

(6) The 3rd Order Polynomial:

$$y_{ij} = \beta_0 + \beta_1 t_{ij} + \beta_2 t_{ij}^2 + \beta_3 t_{ij}^3 + \varepsilon_{ij} \quad j = 1,2,....k\ ; i = 1,2....n$$

where y_{ij} represents weight of child i at measurement occasion j; t_{ij} represents age of child i at measurement occasion j; k = 10 for weight models; and k = 9 for height models.

Appendix 2: Parameter estimates, standard errors and confidence intervals for growth models fitted to weight and height

		Weight			Height		
Model	Coefficient	Estimate	SE	95% CI	Estimate	SE	95% CI
Berkey-Reed 1st order	Intercept	7.31	0.177	(6.96, 7.66)	43.15	0.962	(41.27, 45.04)
	age	0.20	0.003	(0.19, 0.21)	0.37	0.005	(0.36, 0.38)
	Ln(age)	–0.37	0.069	(–0.51, –0.24)	9.90	0.296	(9.32, 10.48)
	1/age	–0.006	0.0006	(–0.008, –0.005)	15.17	2.256	(10.75, 19.59)
	Females	–0.53	0.092	(–0.71, –0.34)	–1.67	0.315	(–2.29, –1.06)
	Female*age	0.010	0.004	(0.002, 0.017)	0.02	0.005	(0.01, 0.03)
Count	Intercept	3.88	0.091	(3.70, 4.05)	45.99	0.377	(45.26, 46.73)
	Age	0.18	0.003	(0.17, 0.19)	0.38	0.004	(0.37, 0.39)
	Ln(age+1)	0.94	0.037	(0.87, 1.01)	9.02	0.123	(8.78, 9.26)
	Females	–0.44	0.096	(–0.71, –0.32)	–1.67	0.314	(–2.29, –1.06)
	Female*age	0.01	0.004	(0.001, 0.017)	0.02	0.005	(0.01, 0.03)
Jenns-Bayley	Intercept	5.36	0.066	(5.23, 5.50)	79.89	0.511	(78.88, 80.90)
	Age	0.21	0.003	(0.207, 0.215)	0.47	0.005	(0.46, 0.48)
	Exp(const)	–6.20	0.006	(–6.21, –6.19)	3.14	0.017	(3.10, 3.17)
	Exp(age)	0.008	0.044	(–0.079, 0.095)	–0.049	0.002	(–0.053, –0.045)
	Females	–0.46	0.102	(–0.66, –0.26)	–1.49	0.323	(–2.13, –0.86)
	Female*age	0.01	0.004	(0.001, 0.016)	0.02	0.005	(0.01, 0.03)
Adapted Jenss-Bayley	Intercept	5.25	0.082	(5.09, 5.41)	66.89	0.468	(65.97, 67.81)
	Age	0.22	0.003	(0.21, 0.23)	0.75	0.012	(0.72, 0.77)
	$(\text{Age})^2$	–0.000 1	0.000 02	(–0.000 1, –0.000 02)	–0.001	0.000 1	(–0.001 6, –0.001 3)
	Exp(const)	–33.00	0.011	(–33.02, –32.98)	2.76	0.047	(2.76, 2.86)
	Exp(age)	0.14	1.399	(–2.61, 2.89)	–0.18	0.019	(–0.22, –0.14)
	Females	–0.53	0.102	(–0.73, –0.34)	–1.51	0.318	(–2.14, –0.89)
	Female*age	0.01	0.003	(0.002, 0.016)	0.02	0.005	(0.01, 0.03)

2nd Order Polynomial	Intercept	5.28	0.083	(5.12, 5.45)	62.37	0.258	(61.86, 62.88)
	Age)	0.21	0.004	(0.20, 0.22)	0.88	0.007	(0.86, 0.89)
	$(Age)^2$	–0.000 07	0.000 02	(–0.0001, –0.00003)	–0.002	0.000 04	(–0.002 4, –0.002 2)
	Females	–0.54	0.107	(–0.75, –0.33)	–1.63	0.343	(–2.30, –0.95)
	Female*age	0.01	0.004	(0.002, 0.018)	0.02	0.005	(0.01, 0.03)
3rd Order Polynomial	Intercept	4.34	0.081	(4.18, 4.50)	59.47	0.268	(58.95, 59.91)
	Age	0.38	0.006	(0.37, 0.39)	1.17	0.012	(1.15, 1.19)
	$(Age)^2$	–0.004	0.000 1	(–0.004, –0.0036)	–0.007 9	0.000 2	(–0.008, –0.007)
	$(Age)^3$	0.000 02	6e-06	(0.000 019, 0.000 022)	0.000 03	1e-06	(0.000 027, 0.000 031)
	Females	–0.53	0.101	(–0.73, –0.33)	–1.65	0.325	(–2.29, –1.02)
	Female*age	0.01	0.003	(0.002, 0.016)	0.02	0.005	(0.01, 0.03)
